# Standardized Grape (*Vitis vinifera* L.) Extract Improves Short- and Long-Term Cognitive Performances in Healthy Older Adults: A Randomized, Double-Blind, and Placebo-Controlled Trial

**DOI:** 10.3390/foods13182999

**Published:** 2024-09-22

**Authors:** Fabio Amone, Amelia Spina, Anna Perri, Danilo Lofaro, Vincenzo Zaccaria, Violetta Insolia, Chiara Lirangi, Francesco Puoci, Vincenzo Nobile

**Affiliations:** 1 R&D Department, Nutratech S.r.l., 87036 Rende, CS, Italy; fabio.amone@nutratechtesting.com (F.A.); amelia.spina@nutratechtesting.com (A.S.); 2 Department of Experimental and Clinical Medicine, University of Catanzaro “Magna Græcia”, 88100 Catanzaro, CZ, Italy; anna.perri@unicz.it; 3 de-Health Lab, Department of Mechanical, Energy, Management Engineering, University of Calabria, 87036 Rende, CS, Italy; 4 R&D Department, Bionap S.r.l., 95032 Belpasso, CT, Italy; 5 Alma Mater Europea, 6000 Koper, Slovenia; violetta.insolia@almamater.si; 6 Psychologist, 87043 Bisignano, CS, Italy; 7 Department of Pharmacy, Health and Nutritional Sciences, University of Calabria, 87100 Cosenza, CS, Italy; 8 R&D Department, Complife Italia S.r.l., 27028 San Martino Siccomario, PV, Italy

**Keywords:** polyphenols, cognitive function, selective attention, clinical trial

## Abstract

Cognitive decline, a common consequence of aging, detrimentally affects independence, physical activity, and social interactions. This decline encompasses various cognitive functions, including processing speed, memory, language, and executive functioning. This trial aimed to investigate, with a double-blind, placebo-controlled clinical trial on 96 healthy older adults, the efficacy of once-daily 250 mg of a standardized grape (*Vitis vinifera* L.) juice extract (Cognigrape^®^) in improving short- and long-term cognitive functions. The results revealed significant improvements across multiple cognitive domains, notably immediate and delayed memory, visuospatial abilities, language, and attention, with improvements occurring within just 14 days, which continued to improve after 84 days of supplementation. The extract exhibited statistically significant enhancements in the Mini-Mental State Evaluation (MMSE), assessment of neuropsychological status (RBANS), “Esame Neuropsicologico Breve 2 (ENB-2), and Modified Bells Test (MBT) scores, with the latter test revealing a significant improvement in selective attention within just 90 min of the first dose. These positive results highlight the potential this natural grape extract has on improving cognitive function both acutely and chronically in a healthy aging population, which in turn supports a longer health span, at least cognitively.

## 1. Introduction

Due to the rising life expectancy and the aging of the global population, health issues linked to aging will become more prevalent. This will contribute to the already significant social and economic burden associated with caregiving [1]. The global population has tripled from 2.9 billion in 1950 to 7.8 billion in 2020. Over the past seven decades, average life expectancy increased from 47 to 73 years, expanding by 26 years. However, this remarkable increase in human longevity has led to a disproportionate increase in the number of individuals over the age of 70, posing challenges to global well-being [2].

While longer lifespans offer the opportunity for prolonged relationships and experiences, they also entail increased morbidity, particularly in later life [3]. The gap between lifespan and health span, the period free from disease and disability, is approximately 9 years. Extending the period of good health is a major public health priority since, despite the significant medical, public health, science, and technology advancements, older adults are not necessarily healthier than previous generations.

Cognitive decline, a well-known aging risk factor, impacts independence, physical activity, and social relationships. While longer lifespans allow for more time with family and friends, the gradual loss of cognitive function poses challenges for individuals, their families, and society. Moreover, increased life expectancy correlates with a higher chronic degenerative diseases incidence, including dementia [4]. Cognitive function naturally declines with age, affecting processing speed, attention, memory, language, visuospatial abilities, and executive functioning [5].

Diet, together with lifestyle, is one modifiable factor, proposed by the World Health Organization to optimize intrinsic capacity for healthy aging, including cognitive function, psychological well-being, sensory function, vitality, and mobility and for preventing age-related diseases and maintaining overall health and independence during aging [6,7,8,9,10,11,12,13]. Diet composition and calorie intake are pivotal factors influencing the aging process and age-related ailments [14,15,16,17,18,19]. Diet provides critical protective compounds, including both caloric (such as unsaturated fatty acids) and non-caloric (such as vitamins, minerals, and polyphenols from botanical extracts) nutrients. A balanced diet maintains the body's homeostasis and reduces chronic and degenerative disease risk factors [4,20,21]. Epidemiological evidence suggests that a diet abundant in bioactive compounds can mitigate age-related cognitive decline.

In particular, certain phenolic compounds, renowned for their antioxidant and anti-inflammatory properties, may significantly improve health span, help the body in supporting physiological conditions, have the potential to delay the aging process, and positively impact cognitive functions [21,22,23,24]. There are many studies assessing the ability of food-sourced polyphenols to intervene in mental cognitive functions, and among these, grape polyphenols are known to offer different health benefits in this framework [20,25,26,27,28]. In particular, grape juice is mainly characterized by the presence of anthocyanins (i.e., malvidin and cyanidin) and proanthocyanidins which include monomers, oligomers, and more complex polymers; the main proanthocyanidins are catechin, epicatechin, procyanidin B1, and procyanidin B2 [29].

Grape juice consumption, at a daily amount of 200–500 mL, has been correlated with improved cognitive performance in the early stage of mild cognitive decline [20]; moreover, another study on purple grape juice showed an improvement in cognitive performance and mood [30]. Additionally, the supplement of Málaga muscatel raisins (50 g) has also shown long-term (6 months) slight improvements in cognitive performance such as visuospatial/executive capacity, language, orientation, overall Montreal Cognitive Assessment (MoCA) test scores, quality of life, and functional activities [31]. In the aged rat model, it was demonstrated that two concentrations of Concord grape juice (10% and 50%) were able to act on reversing age-related deficits. The results showed that 10% grape juice improved dopamine release and cognitive performance, while 50% grape juice enhanced motor function [32]. Positive outcomes have also been assessed in a long-term evaluation through a double-blind placebo-controlled clinical trial involving 111 healthy older adults, where a commercial grape extract supplementation (Cognigrape^®^) for 12 weeks resulted in increased cognitive performance, improved mood, and enhanced overall neuropsychological condition [27].

This trial aimed to confirm the long-term activity already described in the literature [27], and to investigate, for the first time, the immediate and early-term activity of a standardized grape extract, using the most recognized and scientifically approved tests.

## 2. Materials and Methods

### 2.1. Trial Design

The trial was a single-center, randomized (1:1 balanced randomization), double-blind, placebo-controlled, parallel group trial, conducted in a single site at Nutratech S.r.l. (a Complife company), Rende, Italy, between July 2022 and March 2023. It consisted of a screening visit followed by a baseline visit (D0) and three follow-up visits during an 84-day supplementation period. During the baseline visit, the product efficacy was tested in the short term, after 90 min from a single product intake. Eligible subjects were randomized into the study at baseline (visit 1, D0). The study endpoints included the Mini-Mental State Evaluation (MMSE), Repeatable Battery for the Assessment of Neuropsychological Status (RBANS), a complete neuropsychological battery (“Esame Neuropsicologico Breve 2”, ENB-2), and the Modified Bells Test (MBT). Apart from the MBT that was conducted at baseline and 90 minutes (D0+90min) after a single product intake, all the other test batteries were conducted at baseline, at visit 2 (week 2, D14), at visit 3 (week 4, D28), and at visit 4 (week 12, D84). All the trial procedures were carried out in accordance with the World Medical Association’s (WMA) Helsinki Declaration and its amendments. The study protocol and all the trial-related documents were approved by the “Comitato Etico di Ateneo (CEA) Università della Calabria” (ref. no. 0050243 by 29.06.2022). The trial was registered at ISRCTN registry, number ISRCTN10100061, https://doi.org/10.1186/ISRCTN10100061, accessed on 18 September 2024.

### 2.2. Participants

Eligible subjects were all healthy males and females aged more than 55 years old with normal cognitive function. Subjects suffering from mental disorders (both medicated and unmedicated) were not eligible to participate in the trial. Before the inclusion in the trial, interested subjects were administered the MMSE test for the assessment of their cognitive functioning. An MMSE score ≥ 24 was required for enrollment. Subjects with an MMSE score ≥ 24 and who fulfilled all inclusion and exclusion criteria were asked to participate in the clinical study and were scheduled for their inclusion visit (visit 1). Exclusion criteria were any disorder or therapy (drugs or food supplements) liable to interfere with the treatment under study, smokers, body mass index (BMI) above 30, pregnant or breastfeeding women, excessive alcohol consumption (>5 drinks per week), history of drug, alcohol, and other substance abuse, known food intolerance or food allergy, involvement in a clinical or food study within the previous month, unstable medical diseases (cardiac arrhythmias or ischemia, uncontrolled hypertension and hypotension, diabetes mellitus, kidney failure), history of paralysis or cerebral vascular accident, active cancers or chemotherapy, treatment with selective serotonin reuptake inhibitors (SSRIs), and other factors limiting the volunteer ability to cooperate during the study. The trial further excluded subjects not using the active/placebo supplement for more than 1 week. Any intake of memory-improving drugs or food supplements that can interfere with the central nervous system (CNS) activity and/or the consumption of foods or beverages enriched in polyphenols 24 hours before each visit was prohibited.

### 2.3. Interventions and Randomization 

Subjects were assigned randomly to the active or the placebo products treatment arm in a 1:1 ratio. The randomization list was generated by an external statistician using the “Efron’s biased coin” algorithm (PASS 11, version 11.0.8, PASS, LLC, Kaysville, UT, USA). The active treatment arm received (daily) one capsule containing 250 mg of a commercially available (Cognigrape^®^, Bionap S.r.l., 95032 Piano Tavola Belpasso, CT, Italy) extract of *Vitis vinifera* (L.) supported on maltodextrins [30–40%], pregelatinized corn starch (87.75 mg), vegetable magnesium stearate (1.35 mg), talc (0.45 mg), and colloidal silica (0.45 mg). The active ingredient is a spray-dried standardized powder extract from red grape juice containing concentrated active grape substances such as proanthocyanidins (>9%, *w*/*w*) and anthocyanins (4–5% as malvidin-3-glucoside, *w*/*w*). The placebo arm received one capsule daily having the same appearance of the active product and containing maltodextrin (250 mg), pregelatinized corn starch (87.75 mg), vegetable magnesium stearate (1.35 mg), talc (0.45 mg), and colloidal silica (0.45 mg). The study was double-blind, and neither the subjects nor the personnel involved in the study were aware of the active/placebo distribution list. Any unused product was returned by the subjects and was used to assess compliance to treatment.

### 2.4. Outcomes

The Italian [33] version of the Mini-Mental State Evaluation (MMSE) [34] was used to assess cognitive function impairment at baseline and throughout the study. The MMSE is a simple questionnaire composed of very simple questions and graphical works, allowing to examine functions including orientation (to time and to place), registration (repeating named prompts), attention and calculation, recall, language, repetition, and complex commands. The maximum score for MMSE is 30 and is corrected for age and scholarity. A score of 25 is classed as normal, while a score below 24 is indicative of possible cognitive impairment [35,36].

For the assessment of the improvement of cognitive function, the Italian version [37] of the Repeatable Battery for the Assessment of Neuropsychological Status (RBANS) was used. The RBANS consists of twelve subtests which give five scores, one for each of the five domains tested (immediate memory, visuospatial/constructional, language, attention, and delayed memory) [38]. The RBANS global cognition mean score is 100 ± 15 on a scale ranging from 40 to 160, with lower scores indicating worse performance [38,39]. The improvement of the cognitive function in different domains was assessed by “Esame Neuropsicologico Breve 2” (ENB-2) [7]. ENB-2 domains included the following: attention, memory, executive functions, and perceptive and praxis abilities [40,41].

The Modified Bells Test (MBT) [42] was used to assess selective attention. During the MBT the subjects would sit in front of the examiner. First, a “demonstration” sheet is presented to the subject and the examiner gives a demonstration of the task to be performed, cancelling a bell among all the other distractors (e.g., houses, trees, horses, fishes, etc.). The examiner (psychologist) then asked the subject to cross out all the bells on the A4 paper as fast as possible and to ignore the other figures. The total number of sheets administered is four. The time to complete the task is 2 min per sheet. The total number of bells cancelled in the first 30 s is counted. If the subject finished before all targets were detected, the examiner gave only one encouragement, asking, “Are you sure that all bells are now circled?” as reported in the original paper by Gauthier et al. [43]. The result represents the average number of bells cancelled in the first 30 min for each sheet.

### 2.5. Statistical Methods

The sample size was calculated considering similar studies carried out on polyphenols [30,44,45,46,47,48,49,50,51]. Based on these studies, a sample size of 40 (active) + 40 (placebo) subjects was enough to assess the efficacy of the ingredient. A total of 120 subjects were enrolled to consider an anticipated drop-out rate or lower compliance to treatment of 30%.

This paper reports the results of the per-protocol (PP) population including all the randomized subjects with complete data for all the endpoints and compliance to treatment above 90% (e.g., less than 1 week of product use discontinuation). 

Since the data, of all the investigated parameters, were not normally distributed, non-parametric tests were used. The intragroup (variation over time) statical analysis was carried out on raw data by Kruskal–Wallis One-Way ANOVA on Ranks followed by a Tukey–Kramer’s or Wilcoxon signed-rank post hoc test, while the intergroup statistical analysis (active vs. placebo) was carried out on variations vs. baseline by the Mann–Whitney U Test. All the statistical analyses were one-tailed at a 5% significance level (*p* < 0.05). All the statistical analyses were conducted by NCSS 10 (version 10.0.7 for Windows; NCSS, Kaysville, UT, USA) running on Windows Server 2008 R2 Standard SP1 64-bit edition (Microsoft, WA, USA). The level of significance was reported as follows: * *p* < 0.05, ** *p* < 0.01, and *** *p* < 0.001.

## 3. Results

### 3.1. Trial Population

**Figure 1:**
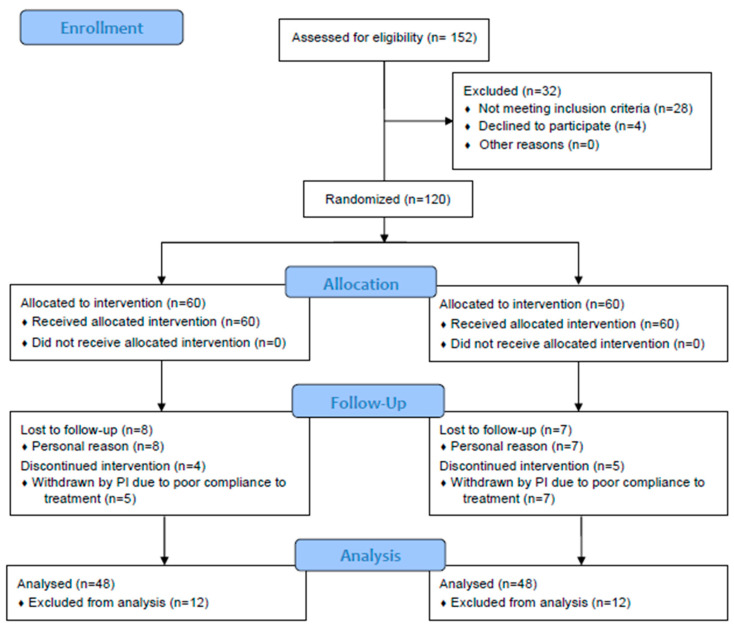
Participant flow chart. Abbreviations: PI, principal investigator.

One hundred and fifty-two subjects were screened for eligibility; out of them, 28 did not meet the inclusion criteria and four declined to participate (Figure 1). The trial randomized then one hundred and twenty subjects; sixty were allocated to the active treatment group and 60 were allocated to the placebo treatment group. The PP population consisted of 96 subjects. Both in the active and in the placebo group 48 subjects finished the study. The reasons for not being included in the PP population were one of the following: withdrew due to personal reasons (n = 8 in the active group and n = 7 in the placebo group) and intake of <90% of the study product (n = 4 in the active group and n = 5 in the placebo group). The ratio between males/females was equal in both the active (33.3% males and 66.7% female) and in the placebo treatment arm (35.4% males and 64.6% female). The mean age (mean ± SE) was 60.7 ± 0.7 in the active group and 60.1 ± 0.7 in the placebo group. Other demographic and baseline data at inclusion are reported in Table 1.

### 3.2. Selective Attention

Ninety minutes after the first intake of the active product, the number of bells found during the MBT increased from 28.8 ± 0.3 (baseline) to 31.8 ± 0.2. This increase was statistically significant (*p* = 0.001) compared to both the baseline and the placebo. When compared to the total number of bells in the sheet, the rate of bells cancellation was 82.4% at baseline and 91.1% after 90 minutes from product intake. This variation corresponds to a statistically significant (*p* = 0.001) improvement by +8.8%. In the placebo group, the variation in the number of bells found was also statistically significant (*p* = 0.001) even if smaller (+4.9%) compared to the baseline. The data of the MBT are reported in Table 2.

### 3.3. Cognitive Function

**Figure 2:**
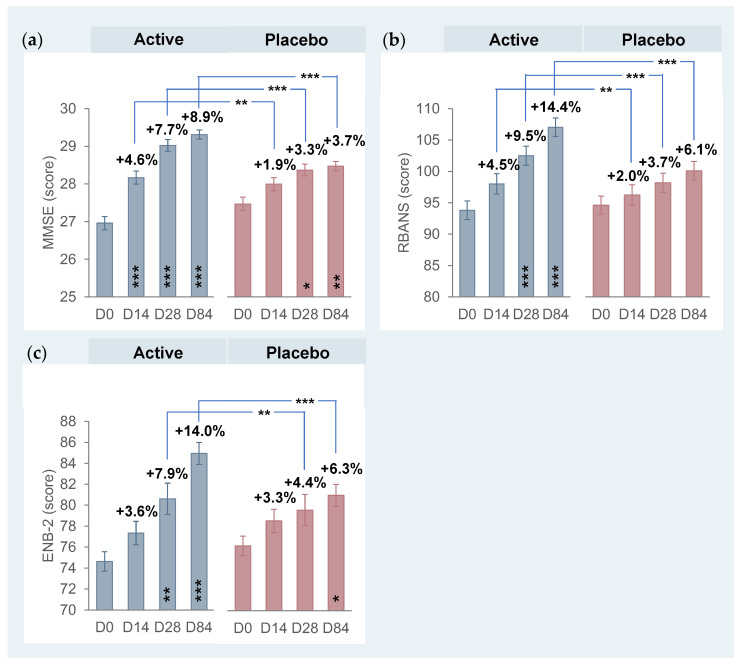
Cognitive function at baseline and after 84 days of product use. (**a**) Mini-Mental State Evaluation (MMSE). (**b**) Repeatable Battery for the Assessment of Neuropsychological Status (RBANS). (**c**) “Esame Neuropsicologico Breve 2” (ENB-2). Data are expressed as means ± SE. The intragroup statistical analysis is reported inside the bars while the intergroup statistical analysis is reported above the bars (blue lines). * *p* < 0.05, ** *p* < 0.01, *** *p* < 0.001.

The cognitive function was assessed by the Mini-Mental State Evaluation (MMSE), Repeatable Battery for the Assessment of Neuropsychological Status (RBANS), and “Esame Neuropsicologico Breve 2” (ENB-2). The results are reported in Figure 2.

During the screening visit the **MMSE** was used to enroll subjects with normal cognitive function (MMSE score ≥ 24). The MMSE score at baseline was 27.0 ± 0.2 in the active treatment arm and 27.5 ± 0.2 in the placebo treatment arm. The total MMSE score in the active treatment arm was significantly increased by +4.6% (28.2 ± 0.2) after just 14 days of supplementation, further improving 7.7% (29.0 ± 0.2), and 8.9% (29.3 ± 0.1) after 28 and 84 days, respectively (all *p* < 0.001 vs. baseline). An increase in the MMSE score was reported in the placebo treatment arm after 28 days (*p* < 0.05) and 84 days (*p* < 0.01). The improvements in total MMSE scores were significantly greater with Cognigrape^®^ than placebo at all time points (i.e., *p* < 0.01 at 14 d and *p* < 0.001 after 28 d + 84 d).

**RBANS** was administered to assess cognitive function in the following domains: immediate memory, visuospatial/constructional abilities, language, attention, and delayed memory. The RBANS score at baseline was 93.8 ± 1.4 in the active treatment arm and 94.5 ± 1.7 in the placebo treatment arm. The total RBANS score in the active group was improved significantly by 4.5% (98.8 ± 1.6) at D14 (*p* < 0.001), while continuing to improve by 9.5% (102.5 ± 1.5) and 14.4% (107.0 ± 1.4) after 28 and 84 days, respectively. This increase was statistically significant compared to the placebo group after 28 (*p* < 0.001) and 84 (*p* < 0.001) days of supplementation. A smaller, trending increase (*p* > 0.05) was seen in the placebo treatment arm. The change in RBANS scoring between active and placebo treatment arms was statistically significant at D28 (*p* < 0.01) and D84 (*p* < 0.001). When analyzed separately, the active treatment significantly positively influenced each of the five domains of RBANS (Table 3). Immediate memory improved 11.2% after 14 days (*p* < 0.05 vs. D0), which was not statistically significant when compared to placebo. By D28 and D84, immediate memory improved 16.0% and 12.8%, respectively, which were significant vs. both baseline and placebo (*p* < 0.05). At D28, visuospatial/constructional abilities improved 18.1% (*p* < 0.05 vs. D0) as well as language 24.2% (*p* < 0.01 vs. D0) and attention 15.9% (*p* < 0.05 vs. D0). After 84 days of treatment, improvements in all the cognitive domains were significantly greater than that seen with placebo, which produced no significant increases in any of the five cognitive domains measured by RBANS.

The cognitive function was further investigated by **ENB-2**. The total ENB-2 score at baseline in the active group was 74.6 ± 0.9 and 76.1 ± 1.1 in the placebo group. The increase in total ENB-2 score in the active treatment arm was by +3.6% (77.3 ± 1.1), +7.9% (80.6 ± 1.5), and +14.0% (84.9 ± 1.0) after 14, 28, and 84 days, respectively. Similar to what happened for RBANS, this increase was statistically significant compared to baseline after 28 (*p* < 0.01) and 84 (*p* < 0.001) days of supplementation. A trend towards improvement was seen with placebo, while the variation was statistically significant only after 84 days of intake (*p* < 0.05). The variation in ENB-2 scoring between active and placebo treatment arms was statistically significant at D28 (*p* < 0.01) and D84 (*p* < 0.001). When analyzed separately, the active treatment significantly positively influenced almost all the cognitive domains of **ENB-2** (Table 4). The improvement in digit span (+22.9%), immediate recall prose memory (+15.2%), and copy drawing test (+28.8%) occurring after just 14 days of Cognigrape^®^ supplementation were significant compared to placebo. At D28 a significant improvement vs. placebo was observed in the active treatment group for the digit span (+31.0% vs. +3.4%, *p* < 0.01), immediate recall prose memory (+22.6% vs. +5.5%, *p* < 0.05), delayed recall prose memory (+16.5% vs. +5.8%, *p* < 0.05), interference memory at 30 s (+14.0% vs. +4.9%, *p* < 0.05), spontaneous drawing test (+40.6% vs. 20.8%, *p* < 0.05), and copy drawing test (+46.5% vs. 23.6%, *p* < 0.05). At the end of treatment (D84), all the cognitive domains, except for the abstract reasoning test, were statistically significantly improved with most of the variation in the cognitive domains being statistically improved when compared to the placebo treatment. In the placebo treatment arms and compared to baseline, significant improvements were seen at D84 in the trial-making test, part A and the trial-making test, part B.

## 4. Discussion

The present study aimed to demonstrate the activity of a standardized grape (*Vitis vinifera* L.) juice extract containing anthocyanins and proanthocyanidins across multiple cognitive domains compared to placebo, suggesting for the first time its potential efficacy in enhancing and supporting cognitive functions in the short term and confirming the long-term activity. The overall cognitive improvement was observed through the MMSE, RBANS, ENB-2, and MBT within immediate and delayed memory, visuospatial and constructional abilities, language, and attention and concentration. 

Anthocyanins and proanthocyanidins, polyphenolic compounds found in various foods such as berries, grapes, and other plants, have garnered interest for their potential health benefits, including cognitive function. Literature data suggest that these molecules may exert positive effects on the brain and cognition. Research indicates that these active compounds coming from grapes might offer neuroprotective effects by reducing inflammation and oxidative damage in the brain, could have a positive impact on the peripheral neurovascular system, and could act on the modulation of synaptic plasticity. All these factors contribute to the improvement in cognitive function and protection against cognitive decline associated with aging and neurodegenerative conditions [4,27,53,54,55]. 

Proanthocyanidins found in the tested active group are monomers and dimers. From the literature data, it is known that they are absorbed into the blood even at relatively low levels and can cross the blood–brain barrier (BBB). In contrast, proanthocyanidins larger than trimers, such as those present in grape seed extracts or other plants, are not absorbed [56]. Therefore, their activity on cognition might be related to their ability to be absorbed and be active in situ, exerting their well-known activity as an anti-inflammatory compound and antioxidant with neuroprotective effect [54].” with “in situ, exerting their well-known activity as an anti-inflammatory and antioxidant compound with neuroprotective effect [54]. 

These compounds have been associated with improvements in memory, attention, and other cognitive domains [30]. It has been demonstrated through in vitro and in vivo animal studies that grape anthocyanin-rich diets can enhance cognitive performance and protect against age-related cognitive decline [24,25]; moreover, in human trials a long-term efficacy was demonstrated [20,27,29,30,31,32]. The concentration of the active compounds in grape and grape juice, however, could vary according to environmental factors and grape cultivars, and in turn, the entity of the physiological effects. To decrease the natural variability of the chemical active components in grape juice, the use of standardized extracts containing a fixed amount of anthocyanins and proanthocyanidins could be a good strategy to have reproducible clinical results linked to daily consumption of actives.

The results of the present clinical trial are in line with those described in the literature [4,27,31,57] linking food-based anthocyanin consumption in human intervention trials with long-term cognitive function improvements: the cognitive domains that appear to be acutely sensitive are verbal learning and memory, whereas, in longer-term consumption, they are attention and working memory. The extract tested was demonstrated to be effective within 14 days (first checkpoint) with a statistically significant increase in MMSE score by +4.6%, +7.7%, and +8.9% after 28 and 84 days, respectively, compared to baseline and placebo. The total RBANS score was significantly increased starting from 14 days (+4.5%) compared to placebo and all domains showed a positive increase trend within the short and long term (+9.5% at D28 and +14.4% at D84). The first notable improvements in cognition via RBANS are related to immediate memory, followed by visuospatial/constructional abilities, language, attention, and delayed memory. The cognitive function improvements in the short and long term were also confirmed by the ENB-2 test, for which the score registered an increase in the active group by +3.6%, +7.9%, and +14.0% after 14, 28, and 84 days, respectively. When analyzed separately, the active treatment significantly positively influenced almost all the cognitive domains of ENB-2 having a statistically significant improvement in the short term for the digit span, immediate and delayed recall prose memory, interference memory at 30 s, spontaneous drawing test, and copy drawing test. At the end of the treatment, the active group had long-term statistically significant results (84 days) for all the cognitive domains measured by ENB-2, except for the abstract reasoning test.

Moreover, an acute effect was observed in selective attention which improved 8.8% within 90 min of ingesting the first dose of Cognigrape^®^, an improvement which was highly significant compared to placebo (*p* = 0.001).

It is important, however, to note that research in this area is still ongoing, and results present in the literature may vary between studies, active compounds concentration, dosage of use, and starting raw materials, which highlights the importance of a standardized extract that has a consistent composition of active compounds (such as anthocyanins and proanthocyanidins), as in the case of Cognigrape^®^, which in turn increases the chance of producing reproducible results clinically. The present study is the second clinical trial conducted on this ingredient and the outcomes and results were comparable with the first randomized placebo-controlled clinical trial in which only the long-term results were analyzed and studied [27].

In conclusion, 250 mg/day of Cognigrape^®^ supplementation improved cognition in healthy older men and women (~60 years old) within just 90 min of the first dose as well as after just 14 days with greater improvements seen after 28 and 84 days, thereby demonstrating both acute and chronic improvements in cognition. Improving cognition in healthy older adults lays the foundation for a longer healthier lifespan (i.e., health span), decreasing the gap between life expectancy and the health lifespan in the aging population and alleviating cognitive problems.

## Figures and Tables

**Table 1:** Demographic and baseline data. Data are means ± SE. The percentage of subjects is reported in the parentheses.

	Active (n = 48)	Placebo (n = 48)	*p*-Value
Sex			
Male	16 (33.3%)	17 (35.4%)	n.a.
Female	32 (66.7%)	31 (64.6%)	n.a.
Age	60.7 ± 0.7 [min 56; max 72]	60.1 ± 0.7 [min 56; max 74]	0.5345
Cognitive function			
MMSE	27.0 ± 0.2	27.5 ± 0.2	n.d.
RBANS	93.1 ± 1.4	94.0 ± 1.7	n.d.
ENB-2	74.6 ± 0.9	76.1 ± 1.1	n.d.
Selective attention			
MBT	28.8 ± 0.3	30.2 ± 0.2	n.d.

Note: MMSE, Mini-Mental State Evaluation; RBANS, Repeatable Battery for the Assessment of Neuropsychological Status; ENB-2, “Esame Neuropsicologico Breve 2”; MBT, Modified Bells Test; n.a., not applicable; n.d., not determined according with the CONSORT statement recommendation to not test baseline differences in randomized controlled trials (RCTs) [52].

**Table 2:** Modified Bells Test results. ^a^ Statistically significant vs. D0 (*p* = 0.001); ^b^ statistically significant vs. placebo (*p* = 0.001).

	Active (n = 48)	Placebo (n = 48)
D0	28.8 ± 0.3 ^a^	30.2 ± 0.2 ^a^
D0+90 min	31.8 ± 0.2 (+8.8% ^b^)	31.9 ± 0.2 (4.9%)

**Table 3:** **RBANS cognitive domains**. Data are reported as difference in means ± SE and percentage variation (in brackets).” with “Data are reported as means ± SE and percentage variation (in brackets). Near the mean value is reported the intragroup (vs. baseline) statistical analysis, while near the percentage variation is reported the intergroup (active vs. placebo) statistical analysis. * *p* < 0.05; ** *p* < 0.01; *** *p* < 0.001.

	Active (n = 44)				Placebo (n = 44)			
	D0	D14	D28	D84	D0	D14	D28	D84
Immediate memory	86.7 ± 3.1	92.8 ± 3.1 *	94.5 ± 3.2 *	93.8 ± 3.2 *	89.5 ± 3.2	86.1 ± 3.0	82.6 ± 3.4	87.8 ± 3.2
		(+11.2%)	(+16.0% *)	(+12.8% *)		(+2.4%)	(+1.5%)	(+4.5%)
Visuospatial/constructional abilities	83.2 ± 3.2	84.6 ± 3.7	92.6 ± 3.2 *	102.1 ± 3.2 ***	89.2 ± 3.0	89.0 ± 3.5	89.1 ± 3.3	90.3 ± 2.9
		(+8.4%)	(+18.1%)	(+30.2% ***)		(+4.1%)	(+4.1%)	(+5.3%)
Language	78.5 ± 3.6	85.8 ± 3.8	89.6 ± 3.0 **	93.4 ± 3.6 ***	87.8 ± 3.4	88.8 ± 2.8	91.7 ± 3.4	91.3 ± 3.4
		(+20.4%)	(+24.2%)	(+27.3% *)		(+7.8%)	(+7.9%)	(+7.6%)
Attention	85.3 ± 3.4	92.4 ± 3.5	93.2 ± 3.2 *	103.6 ± 3.0 ***	93.3 ± 3.4	92.5 ± 3.6	97.7 ± 3.5	91.0 ± 3.0
		(+15.6%)	(+15.9%)	(+28.3% ***)		(+6.0%)	(+11.8%)	(+1.5%)
Delayed memory	135.8 ± 1.3	136.1 ± 1.4	135.6 ± 1.0	138.9 ± 1.2 *	135.8 ± 1.5	133.6 ± 1.7	132.8 ± 2.0	134.8 ± 1.7
		(+0.5%)	(+0.2%)	(+2.5% *)		(−1.3%)	(−2.0%)	(−0.6%)

**Table 4:** **ENB-2 cognitive domains**. Data are reported as difference vs. baseline and percentage variation (in brackets). Near the difference is reported the intragroup (vs. baseline) statistical analysis, while near the percentage variation is reported the intergroup (active vs. placebo) statistical analysis. * *p* < 0.05; ** *p* < 0.01; *** *p* < 0.001.

	Active (n = 48)				Placebo (n = 48)			
	D0	D14	D28	D84	D0	D14	D28	D84
01 Digit span	5.5 ± 0.2	5.9 ± 0.1	6.3 ± 0.1 ***	6.7 ± 0.1 ***	5.9 ± 0.2	5.9 ± 0.1	6.1 ± 0.2	6.2 ± 0.2
		(+22.9% **)	(+31.0% **)	(+40.9% ***)		(−1.0%)	(+3.4%)	(+4.9%)
02 Im. rec. pr. mem.	19.6 ± 0.5	20.9 ± 0.4	22.2 ± 0.4 ***	23.3 ± 0.4 ***	20.8 ± 0.5	21.1 ± 0.5	21.9 ± 0.5	22.1 ±0.6
		(+15.2% *)	(+22.6% *)	(+29.2% **)		(+1.3%)	(+5.5%)	(+6.6%)
03 Del. rec. pr. mem.	20.0 ± 0.5	20.9 ± 0.4	22.3 ± 0.5 **	23.4 ± 0.4 ***	20.8 ± 0.6	21.4 ± 0.6	21.9 ± 0.5	22.2 ± 0.6
		(+9.5%)	(+16.5% *)	(+23.0% **)		(+2.8%)	(+5.8%)	(+7.5%)
04 Interf. mem. @10 s	6.6 ± 0.2	6.8 ± 0.1	7.2 ± 0.2 *	7.6 ± 0.1 ***	6.8 ± 0.2	6.9 ± 0.2	7.1 ± 0.2	7.2 ± 0.2
		(+5.6%)	(+11.5%)	(+17.4% **)		(+1.5%)	(+4.9%)	(+5.8%)
05 Interf. mem. @30 s	6.5 ± 0.1	6.9 ± 0.1	7.3 ± 0.2 **	7.6 ± 0.1 ***	6.8 ± 0.2	6.9 ± 0.2	7.1 ± 0.2	7.2 ± 0.2
		(+6.9%)	(+14.0% *)	(+18.6% **)		(+1.3%)	(+4.9%)	(+6.9%)
06 Trial mak. test A	55.4 ± 1.6	51.6 ± 1.3	47.8 ± 1.4 ***	42.8 ± 1.3 ***	55.4 ± 1.9	51.5 ± 1.9	47.8 ± 1.8 *	43.2 ± 1.8 ***
		(−6.3%)	(−13.3%)	(−22.3%)		(−7.2%)	(−13.9%)	(−22.5%)
07 Trial mak. test B	110.5 ± 2.8	103.9 ± 2.5	96.6 ± 2.5 ***	89.7 ± 2.1 ***	109.9 ± 3.1	103.2 ± 2.8	97.4 ± 2.6 **	90.2 ± 2.4 ***
		(−5.7%)	(−12.2%)	(−18.2%)		(−5.8%)	(−10.9%)	(−17.3%)
08 Token test	3.6 ± 0.1	3.8 ± 0.1	3.9 ± 0.1	4.3 ± 0.1 ***	3.7 ± 0.1	3.8 ± 0.1	4.0 ± 0.1	4.0 ± 0.1
		(+3.8%)	(+8.8%)	(+19.2%)		(+3.5%)	(+10.8%)	(+11.3%)
09 Word ph. fluency	7.6 ± 0.2	7.8 ± 0.2	8.3 ± 0.2	8.7 ± 0.2 **	7.7 ± 0.2	7.9 ± 0.2	8.1 ± 0.2	8.2 ± 0.2
		(+2.7%)	(+8.5%)	(+14.8% **)		(+2.7%)	(+5.1%)	(+6.7%)
10 Abs. reas. test	4.6 ± 0.1	4.7 ± 0.1	4.9 ± 0.1	5.1 ± 0.1	4.5 ± 0.1	4.7 ± 0.1	4.8 ± 0.1	4.9 ± 0.1
		(+2.3%)	(+7.8%)	(+11.7%)		(+4.3%)	(+9.4%)	(+11.6%)
11 Cogn. est. test	3.7 ± 0.1	3.8 ± 0.1	4.0 ± 0.1	4.2 ± 0.1 *	3.7 ± 0.1	3.9 ± 0.1	4.0 ± 0.1	4.1 ± 0.1
		(+3.3%)	(+9.5%)	(+16.0%)		(+4.5%)	(+6.8%)	(+10.3%)
12 Test of ov. fig.	24.0 ± 0.8	24.5 ± 0.8	25.6 ± 0.9	27.0 ± 0.7	24.5 ± 0.7	24.6 ± 0.8	25.6 ± 0.8	25.5 ± 0.7
		(+1.7%)	(+6.6%)	(+13.2% ***)		(+0.8%)	(+4.8%)	(+4.7%)
13 Spont. draw. test	1.3 ± 0.1	1.5 ± 0.1	1.7 ± 0.1 **	1.9 ± 0.1 ***	1.5 ± 0.1	1.5 ± 0.1	1.6 ± 0.1	1.6 ± 0.1
		(+15.6%)	(+40.6% *)	(+56.8% ***)		(+9.4%)	(+20.8%)	(+20.8%)
14 Copy draw. test	1.3 ± 0.1	1.6 ± 0.1 *	1.8 ± 0.1 ***	1.9 ± 0.1***	1.4 ± 0.1	1.5 ± 0.1	1.7 ± 0.1	1.6 ± 0.1
		(+28.8% *)	(+46.5% *)	(+55.9% **)		(+9.0%)	(+23.6%)	(+22.6%)
15 Clock draw. test	8.0 ± 0.3	8.1 ± 0.3	8.6 ± 0.4	9.1 ± 0.4	7.9 ± 0.3	8.2 ± 0.3	8.4 ± 0.3	8.5 ± 0.3
		(+1.1%)	(+7.3%)	(+14.2% **)		(+3.6%)	(+5.7%)	(+7.5%)
16 Praxis test	4.9 ± 0.2	5.0 ± 0.2	5.3 ± 0.2	5.5 ± 0.3	4.9 ± 0.2	5.0 ± 0.2	5.2 ± 0.2	5.1 ± 0.2
		(+2.0%)	(+7.9%)	(+11.3% *)		(+1.6%)	(+6.9%)	(+5.9%)

## Data Availability

The data presented in this study are available on request from the corresponding authors. The data are not publicly available since they are the property of the sponsor of the study (Bionap S.r.l., 95032 Piano Tavola Belpasso, CT, Italy).
